# Associations between Oxytocin Receptor Gene Polymorphisms, Empathy towards Animals and Implicit Associations towards Animals

**DOI:** 10.3390/ani8080140

**Published:** 2018-08-14

**Authors:** Melanie Connor, Alistair B. Lawrence, Sarah M. Brown

**Affiliations:** 1 Scotland’s Rural College (SRUC), West Mains Road, Edinburgh EH9 3 JG, UK; alistair.lawrence@sruc.ac.uk; 2 Roslin Institute, University of Edinburgh, Penicuik EH25 9RG, UK; sarah.brown@ed.ac.uk

**Keywords:** *OXTR*, empathy, implicit associations, human-animal-interaction

## Abstract

**Simple Summary:**

Oxytocin is a hormone which acts as a neurotransmitter has been associated with a wide range of human social behaviours. Single nucleotide polymorphisms (SNPs) within the oxytocin receptor gene (*OXTR*) have been described to be involved with human-human empathy, however little is known about *OXTR* SNPs and human-animal empathy and spontaneous reactions towards animals. This has been investigated in the present study with 161 British students and five extensively studied *OXTR* SNPs. Validated, standardized measures for empathy towards animals and spontaneous reactions towards animals have been employed. Results indicate that females show higher levels of empathy and have more positive reactions towards animals than males. Furthermore, empathy towards animals was associated with the absence of the minor A allele on *OXTR* SNP rs2254298. These results indicate that *OXTRs* play a role not only for human social behaviours but also for human-animal interactions.

**Abstract:**

Oxytocin has been well researched in association with psychological variables and is widely accepted as a key modulator of human social behaviour. Previous work indicates involvement of oxytocin receptor gene (*OXTR*) single nucleotide polymorphisms (SNPs) in human-human empathy, however little is known about associations of *OXTR* SNPs with empathy and affective reactions of humans towards animals. Five *OXTR* SNPs previously found to associate with human social behaviour were genotyped in 161 students. Empathy towards animals and implicit associations were evaluated. A General Linear Model was used to investigate the *OXTR* alleles and allelic combinations along with socio-demographic variables and their influence on empathy towards animals. Empathy towards animals showed a significant association with *OXTR* SNP rs2254298; homozygous G individuals reported higher levels of empathy towards animals than heterozygous (GA). Our preliminary findings show, for the first time, that between allelic variation in *OXTR* and animal directed empathy in humans maybe associated, suggesting that *OXTRs* social behaviour role crosses species boundaries, warranting independent replication.

## 1. Introduction

Human interactions with animals are manifold and it has been shown that human attitudes towards animals, that is, in particular positive attitudes towards animals, are of vital importance to the welfare of animals under human care [1]. It has been shown that attitudes towards animals are influenced by a variety of factors such as socio-cultural and religious norms [2,3], early life experiences [2], personality traits [4], species preference [5,6], the degree of interactions with animals [7] and people’s perceptions of the degree of cognitive and behavioural similarity between humans and animals [8,9] to name a few. This list is not conclusive and attitude research generally shows that only a small amount of variance (between 4–18% [4,10] can be accounted for by a combination of aforementioned attitude modifiers).

Empathy has been suggested to be a strong and reliable predictor predominately for inter-human relationships contributing to an increase of positive attitudes and behaviour [11]. Empathy has been defined as reactions of one individual to the observed experiences of another individual [12] encompassing both affective and cognitive components [13]. Empathy towards other humans has been a main predictor for maintaining pro-social relationships [14] and it has also been shown to improve attitudes to members of stigmatised groups [15]. Likewise, empathy has also been reported to correlate with attitudes towards the treatment of non-human species [4,5,16]. Indeed associations have been found between human-human and human-animal directed empathy suggesting that the two types of empathy measures are in some way linked, although they are unlikely to tap a single unitary construct [17]. The relationships between covariates also suggest different sources of variation between human-human and human-animal empathy scores [17].

Empathy has been shown to influence farmer’s attitudes and behaviour towards dairy cattle [18] and was shown to be positively correlated with milk yield [19]. Veterinarians with higher levels of empathy also showed better pain recognition in cattle [20]. A study investigating dog owners’ empathy and attitudes towards animals and their relationship with pain perception in dogs revealed empathy to be a strong predictor of these pain perceptions [21]. Empathy has therefore been suggested to be an important factor positively influencing human-animal interaction [1]. An experimental study investigating different scenarios of the need for medical attention in an animal or human abuse victim, found that people show at least as much empathy for animals as for humans [22]. These results indicate that empathy for animals might translate into prosocial behaviour towards them.

Human empathy has been linked with pro-social behaviour, social organisation and behaviour [11] in general which are key aspects in the success of many animal species. Social behaviour which can involve both positive and negative interactions is a complex process involving the combined effects of hormones, genetics and past experience (reviewed in [23]). The ability to maintain social relationships has been proposed as a need routed in human evolution with the inability to maintain social relationships, or of feelings of social exclusion, being associated with reduced psychological wellbeing [24]. Oxytocin (OT) a neurohypophyseal hormone, produced in the paraventricular nucleus and excreted by the pituitary is widely accepted as a key modulator to social and emotional behaviour in mammals [25]. Oxytocin’s primary receptor (OXTR) is localized to chromosome 3p25 (in humans) and contains 28 known single nucleotide polymorphisms (SNPs) (https://www.snpedia.com/index.php/OXTR) [26], with various observed effects. Of these 28, 10 of these SNPs have been extensively studied in relation to social behaviour and psychological health in humans (reviewed in [27]). Associations have been recorded at various sites, with 2 *OXTR* SNPs, rs53576 and rs2254298 being particularly prevalent. Allelic variation at rs53579 has been linked to individual differences in pro-social temperament [28], stress reactivity [29], emotional support seeking [30] and loneliness [31]. Variation at rs2254298 has been linked with maternal depression [32], depression and anxiety in adolescence [33]. Furthermore, it has been suggested that *OXTR* is associated with affective behaviours [34] and empathy (inter human empathy) [29] specifically with the sub-scale empathic concern [35] and trait empathy [36]. 

Intranasal administration of oxytocin in humans has been shown to increase trust [37], improve the interpretation of social cues [38] and increase eye-gaze [39] in healthy individuals. However given OTs poor ability to cross the blood brain barrier there is debate over the methodology of these studies and the mode of action of OT in such experiments [40]. The effect of intranasal OT administration is also context and individual dependent (as reviewed in [41]) and it has been proposed that the interaction between individual differences in endogenous OT plasma levels and allelic variations in the oxytocin receptor gene (*OXTR*) may form complex interactions which modulate the effects of exogenously administered oxytocin on an individual basis. For example, the previously mentioned review paper from Bartz et al. (2011) [41] states that, of those studies tested, >40% showed no main effect of OT and >60% reported individual differences as moderators. In some cases intranasal oxytocin actually produced antisocial behaviour traits, such as distrust [42] and envy [43].

While *OXTR* has been well researched in connection with psychological variables within and between humans, little is known about its association with empathy and affective reactions towards non-human species, though the reverse has been studied. In dogs treated with intranasal oxytocin, *OXTR* polymorphisms and their interaction were associated with dogs’ human directed social skills [44,45,46]. However, the effect of OXTR polymorphisms, in the absence of an endogenous primer (such as intranasal OT), on animal directed empathy in humans has thus far not been tested. Based on the aforementioned associations of the *OXTR* with human-human empathy, the present study attempted to explore possible associations of the effect of allelic variation at 5 known SNP loci in *OXTR* (rs2268491, rs13316193, rs4686302, rs2254298, rs53576) and human-animal empathy and implicit associations towards animals. Implicit associations are associations which have been exposed by means of indirect or implicit measures [47]. Traditionally attitudes have been assessed using self-report measures which in turn have been criticised due to influences by uncontrolled extraneous variables like environmental factors, order of items, wording of items, that is, social desirability, especially in regard to sensitive topics [47,48]. Implicit associations tests aim to detect the strength of people’s automatic associations between mental representations of objects [47], that is, have been used in an attempt to access attitudes indirectly [47]. Our specific aim was to investigate whether empathy towards animals and/or implicit associations to animals share some of the same biological basis as for human-human empathy by testing their association with *OXTR* SNPs known to associate with pro-social behaviour in human, hence adding to our understanding of the development of our perceptions and behaviour towards non-human animals. Considering the complexity behind empathy modulation—that is, its novelty in the field of animal welfare—there is scarce evidence that supports the belief that empathy provides another means to improve animal welfare. Since empathy expressions are contextual and affected by empathizer characteristics (e.g., pet-ownership, gender) as well as the relationship between empathizer and target [49], a sample of animal care students and professionals, that is, non-animal care students and professionals were investigated. 

## 2. Methods

### 2.1. Ethics

Sample and study design were approved by the National Health Service (NHS) research ethics committee for healthy volunteers (ACCORD) and the SRUC Research Ethics in Student Projects committee (REC code 14/HV/0005, IRAS project ID 154914). All individuals gave informed consent at the start of the study and were free to withdraw at any time.

### 2.2. Measures

#### 2.2.1. Empathy Measures

Animal Empathy Scale (AES) was measured using the 22-item scale proposed by Paul (2000) [17]. Items were measured on a 6-point Likert scale ranging from I “completely disagree” to “fully agree.” Reversed phrased items were re-coded for the analysis and a mean scale score was computed.

#### 2.2.2. Implicit Associations

**Figure 1:**
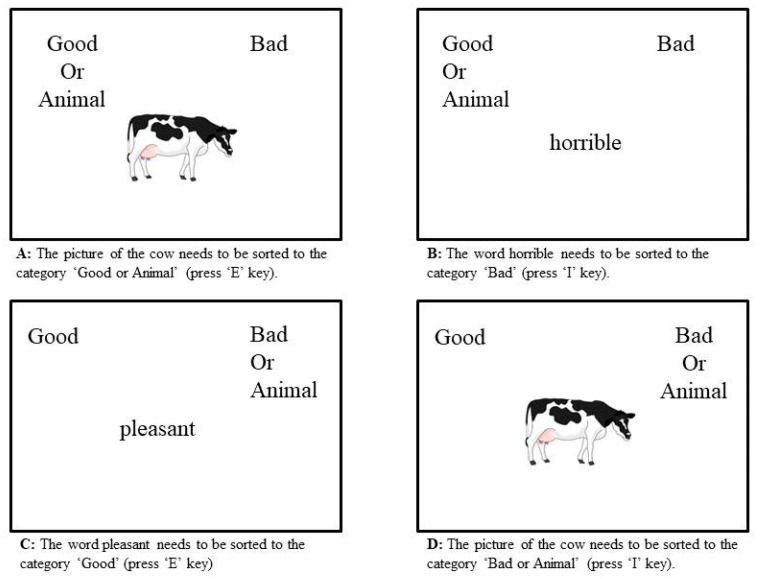
Examples from the IAT representing possible evaluation tasks.

Implicit associations to animals were assessed using a single category picture implicit association test (IAT), which has been adapted from the Karpinski and Steinman [50] IAT measure. The implicit association test is a timed computer-based matching task [51] using Inquisit 4 (2014), Millisecond software. Participants were presented with positive attributes as positive words (celebrating, cheerful, excellent, excitement, fabulous, friendly, glad, glee, happy, laughing, likable, loving, marvellous, pleasure, smiling, splendid, superb, paradise, triumph, wonderful), negative attributes as negative words (angry, brutal, destroy, dirty, disaster, disgusting, dislike, evil, gross, horrible, humiliate, nasty, noxious, painful, revolting, sickening, terrible, tragic, ugly, unpleasant, yucky) and animal pictures. In total 21 words for each attribute category were used along with 12 drawn images of animals (from Sparklebox.co.uk) [52] randomly presented in the middle of the computer screen, below the target categories. Animals used for the IAT included pet animals such as dog, rabbit, budgerigar; British farm animals such as cow, pig and sheep; and British wild animals such as deer and fox. Participants were instructed to use the ‘I’ and ‘E’ key on the keyboard to sort the animals into the target categories depending on the description of the task (task 1: sorting pictures of animals and positive words into the target category ‘Good or Animals’ (Figure 1A); task 2: sorting pictures of animals and negative words into the target category ‘Bad or Animals’ (Figure 1D). An IAT score was generated by the program based on the participant’s latency to respond and error rates. The program automatically records and summarises false responses (when respondents categorized the attributes (words and pictures) to the wrong target category) and too slow responses (>500 ms) [50]. The IAT score corresponds to participant’s implicit associations with the target category in this case animals [50].

### 2.3. Saliva Sampling and DNA Extraction

Participant saliva was collected using the ‘Saliva DNA Collection, Preservation and Isolation Kit’ from Norgen Biotek (Ontario, ON, Canada) as per the manufacturer’s instructions. Participants were asked to fill a collection tube with saliva up to the 2 mL mark and the supplied preservative was then added by the researcher. Samples were agitated for 10 s to mix the saliva and preservative and then stored in a cold room (4–8 °C) until required for DNA extraction.

DNA extraction was performed by ethanol precipitation using reagents provided in the saliva DNA collection, preservation and isolation kit. Proteinase K was added to the sample and incubated at 55 °C for 10 min. Binding buffer B was then added and the mix incubated for a further 5 min at 55 °C. Isopropanol was added and the sample mixed before centrifugation. Supernatant was discarded and 70% ethanol added to the DNA pellet. Sample was left to stand on the bench top for 1 min then centrifuged. Supernatant was then again discarded and pellet rehydrated in TE buffer. Sample was incubated at 55 °C for 5 min and then stored at −20 °C until required for sequencing.

### 2.4. SNP Sequencing

182 DNA samples were submitted for sequencing. Four samples were run in duplicate as internal controls. Sequencing of SNPs was carried out by LGC Genomics Ltd. (Middlesex, UK). One of the authors provided flanking sequencing for primer design taken from Ensembl. Every sample was sequenced for each of the 5 SNPs. Where a read could not be obtained the SNP was called as 0, otherwise SNPs were called as per regular annotation. 68.7% of samples tests (125/182) returned a result for all 5 SNPs and 1.65% (3/182) of samples tested failed to return a result for any SNP. Further analysis was only performed on SNPs from those individuals for whom an IAT score and an AES score could be calculated (*n* = 161). The summary table (Table 1) below outlines the success rate for each individual SNP used in the analysis.

### 2.5. Statistics

Data were analysed using IBM SPSS Statistics V22.0. To assess the reliability of each scale Cronbach’s alpha was computed which represents a measure of the internal consistency of items forming a scale. The empathy measure and IAT measures were analysed using general linear models (GLMs) fitting SNPs rs2268491, rs13316193, rs4686302, rs2254298, rs53576 by presence or absence of the minor allele, gender and working in an animal care profession as fixed effects. Residual plots were examined to confirm normality assumptions for both models were satisfied. The value for statistical significance was set to α = 0.05.

### 2.6. Participants

Participants were recruited from four campuses of a Higher Education Institute (Scotland’s Rural College (SRUC)). Data are presented only for participants for whom an IAT score and an AES score could be calculated in addition to at least one successful SNP assay. In total 161 people met these criteria, 56 (34.8%) were male and 104 (64.6%) female, 1 participants (0.6%) did not report their gender. 97 (60.2%) of the participants reported to study in the animal care profession of which 80.8% (*N* = 84) were female, 46 (28.6%) reported not to study in the care profession and 18 participants did not report as to whether they study in the animal care profession. The mean age of the participants was 22.4 years (SD = 7.97, min = 16 years, max = 60 years). Participants reported to be of British or European white ethnicity (*N* = 117), one participant reported to be black and the rest did not report their ethnicity. Self-reported dietary requirements show that 67.1% (*N* = 108) of the participants reported following an omnivorous diet, while 23% (*N* = 37) reported to following a restricted meat diet avoiding all or some animal products, the rest 9.9% (*N* = 16) did not answer this question. Most, 76.4% (*N* = 123) of the participants reported currently owning a pet, while 85.7% (*N* = 138) had done so in the past.

## 3. Results

### 3.1. Empathy Measures

The Animal Empathy Scale (AES) [17] showed a good overall reliability for the present sample, Cronbach’s α = 0.849 (*N* = 22). The mean empathy towards animals score was 4.3 (SD = 0.62, *N* = 161). Females (M = 4.91, SD = 0.54, *N* = 104) had a significantly higher AES Score than males (M = 3.91, SD = 0.54, *N* = 56), t(158) = −7.08, *p* < 0.001.

In order to test the influence of the allelic combination in *OXTR* single nucleotide polymorphisms (SNPs), *OXTR* SNPs were coded for presence or absence of the minor allele resulting in binary predictor variables [53] (Table 2).

A GLM model was used to investigate the influence of the alleles along with gender, working in an animal care profession, on empathy towards animals (AES). Results show that working in an animal care profession and being female are predictors of empathy towards animals (Table 3). An association with higher empathy towards animals was observed for SNP rs2254298 (Table 3).

### 3.2. Implicit Associations to Animals (IAT)

Participants of the present study had an average IAT score of M = 0.035 (SD = 0.22, *N* = 161). This score did differ significantly from zero (t = 1.98, df = 160, *p* = 0.049). Females (M = 0.07, SD = 0.19, *N* = 104) had a significantly higher IAT Score than males (M = −0.03, SD = 0.25, *N* = 56), t(158) = −2.93, *p* = 0.004.

A GLM model was used to investigate the influence of the alleles along with gender, working in an animal care profession on implicit associations (IAT score). The results found that only rs53576 showed an association with the IAT score (Table 4).

No correlation between implicit associations towards animals and empathy towards animals was found (r = 0.144, *N* = 161, *p* = 0.068).

## 4. Discussion

The present study investigated the effect of allelic variation at 5 SNP loci on the *OXTR* (rs2268491, rs13316193, rs4686302, rs2254298, rs53576) which had previously been shown to associate with human-human empathy and other pro-social traits [28,29,30,31]. The present study investigated if *OXTR* SNPs also associate with empathy to and implicit associations with animals. The animal empathy scale (AES; [17]) we applied showed good internal reliability; Cronbach’s alpha = 0.849 exceeding reported reliabilities of 0.71 [54] and 0.78 [17]. Results of the present study found that females had higher levels of empathy towards animals than male participants a finding previously reported in other work [17,55]. Furthermore, pet ownership and working in an animal care profession were significantly associated with higher levels of empathy towards animals. These results confirm previous results investigating farmers’ [18] veterinarians’ [20] empathy towards animals and their ability to recognize pain in cattle. The scores for the AES also showed a significant association with one of the genotyped SNPs (rs2254298). Homozygous G individuals reported higher levels of empathy towards animals than minor A allele carriers. However as in this sample there were only 2 homozygous A individuals, due to the fact that the present sample comes from a European population where the A allele is quite uncommon (http://grch37.ensembl.org), it is impossible to say if this effect was diluted in heterozygous individuals compared to AA homozygotes. Furthermore, *OXTR* rs2254298 has also been shown to be involved in emotional empathy in schizophrenic and healthy individuals [35]. In order to also investigate implicit associations of animals the present study employed an implicit association task (IAT) to evaluate the effects in dependence of allelic variation. Results show that participants’ (male and female combined) associations with animals were slightly positive indicating positive implicit associations with animals. Furthermore, female participants showed higher IAT scores again indicating that women are more affective and empathetic towards animals [5,17]. The results of the GLM did indicate associations between the predictor variables care profession, rs53576 and the IAT score. Interestingly, results of the present study did not show a great variation in IAT scores, with participants’ implicit associations of animals being assessed as only slightly positive. Further research could investigate a broader sample including non-pet owners and people with differing degrees of attachment or liking of animals and or attachment to pets to account for a greater variation in both implicit measures and explicit measures.

## 5. Conclusions

The study for the first time supports a role for the oxytocin receptor in enabling animal directed empathy in humans. It suggests when taken in the context of the wider literature that the oxytocin receptor may provide a common biological substrate for both human-human and human-animal empathy. The present study using explicit measures of empathy has also confirmed previous work showing that females express higher levels of empathy to animals. In addition, we have shown that other characteristics (working in animal care) are associated with higher human-animal empathy. We also applied for the first time a test of implicit associations to animals and found that whilst the participants in general had neutral associations, again females had more positive associations with animals. 

## Figures and Tables

**Table 1:** Summary of Single nucleotide polymorphism (SNP) calling success rates. Hom (Homozygous) 1 and Hom 2 refer to the alleles as they appear under the SNPs in the table. That is, for rs2268491, C is allele 1 and T is allele 2. Hom 1 individuals at rs2268491 would be homozygous for C and Hom 2 individuals homozygous for T; Het (Heterozygous) individuals would be CT. Success rate of the assay is noted in the final column as the % of samples submitted that returned a read.

SNP	Hom 1	Het	Hom 2	Total	% of Submitted
rs2268491 (C/T)	129	19	1	149	92.5
rs13316193 (C/T)	16	49	55	120	74.5
rs4686302 (C/T)	121	29	1	151	93.8
rs2254298 (G/A)	104	25	2	131	81.4
rs53576 (G/A)	64	51	14	129	80.1

**Table 2:** Distribution of *OXTR* SNPs for the present sample coded as presence or absence of the minor allele.

SNP	Coding	N Male	N Female	Total N
rs2268491	CT/TT (t present)	6	14	20
	CC (t absent)	45	83	128
rs13316193	CT/CC (c present)	25	40	65
	TT (c absent)	19	35	54
rs4686302	CT/TT (t present)	10	20	30
	CC (t absent)	43	77	120
rs2254298	AA/AG (a present)	7	20	27
	GG (a absent)	38	65	103
rs53576	AA/AG (a present)	23	41	64
	GG (a absent)	21	43	64

Note: Total N reflects N of participants who reported their gender (one participant did not report their gender).

**Table 3:** Test of model effects Type I, that is, parameter estimates, dependent variable AES.

Predictor	β	Wald χ^2^ (1)	*p*
Care profession	−0.356	4.97	0.026
Gender	−0.456	8.81	0.003
rs2268491	−0.687	2.61	0.106
rs13316193	0.094	0.74	0.391
rs4686302	−0.211	2.39	0.122
rs2254298	−0.799	4.12	0.042
rs53576	−0.356	0.001	0.972

Note: gender (0 = male, 1 = female), care profession (0 = no, 1 = yes). Coding for SNPs was based on presence or absence of minor allele. *N* = 94.

**Table 4:** Test of model effects, i.e., parameter estimates, dependent variable IAT score.

Predictor	β	Wald χ^2^ (1)	*p*
Care profession	−0.086	2.38	0.123
Gender	−0.020	0.15	0.701
rs2268491	−0.126	0.73	0.392
rs13316193	0.070	3.38	0.066
rs4686302	−0.006	0.02	0.898
rs2254298	0.033	0.06	0.807
rs53576	0.099	6.11	0.013

Note: gender (0 = male, 1 = female), care profession (0 = no, 1 = yes). Coding for SNPs was based on presence or absence of minor allele. *N* = 94.
